# Prediction of infectious disease epidemics via weighted density ensembles

**DOI:** 10.1371/journal.pcbi.1005910

**Published:** 2018-02-20

**Authors:** Evan L. Ray, Nicholas G. Reich

**Affiliations:** 1 Department of Mathematics and Statistics, Mount Holyoke College, South Hadley, Massachusetts, United States of America; 2 Department of Biostatistics and Epidemiology, University of Massachusetts, Amherst, Massachusetts, United States of America; National Institutes of Health, UNITED STATES

## Abstract

Accurate and reliable predictions of infectious disease dynamics can be valuable to public health organizations that plan interventions to decrease or prevent disease transmission. A great variety of models have been developed for this task, using different model structures, covariates, and targets for prediction. Experience has shown that the performance of these models varies; some tend to do better or worse in different seasons or at different points within a season. Ensemble methods combine multiple models to obtain a single prediction that leverages the strengths of each model. We considered a range of ensemble methods that each form a predictive density for a target of interest as a weighted sum of the predictive densities from component models. In the simplest case, equal weight is assigned to each component model; in the most complex case, the weights vary with the region, prediction target, week of the season when the predictions are made, a measure of component model uncertainty, and recent observations of disease incidence. We applied these methods to predict measures of influenza season timing and severity in the United States, both at the national and regional levels, using three component models. We trained the models on retrospective predictions from 14 seasons (1997/1998–2010/2011) and evaluated each model’s prospective, out-of-sample performance in the five subsequent influenza seasons. In this test phase, the ensemble methods showed average performance that was similar to the best of the component models, but offered more consistent performance across seasons than the component models. Ensemble methods offer the potential to deliver more reliable predictions to public health decision makers.

## Author summary

Public health agencies such as the US Centers for Disease Control and Prevention would like to have as much information as possible when planning interventions intended to reduce and prevent the spread of infectious disease. For instance, accurate and reliable predictions of the timing and severity of the influenza season could help with planning how many influenza vaccine doses to produce and by what date they will be needed. Many different mathematical and statistical models have been proposed to model influenza and other infectious diseases, and these models have different strengths and weaknesses. In particular, one or another of these model specifications is often better than the others in different seasons, at different times within the season, and for different prediction targets (such as different measures of the timing or severity of the influenza season). In this article, we explore ensemble methods that combine predictions from multiple “component” models. We find that these ensemble methods do about as well as the best of the component models in terms of aggregate performance across multiple seasons, but that the ensemble methods have more consistent performance across different seasons. This improved consistency is valuable for planners who need predictions that can be trusted under all circumstances.

## Introduction

The practice of combining predictions from different models has been used for decades by climatologists and geophysical scientists. These methods have subsequently been adapted and extended by statisticians and computer scientists in diverse areas of scientific inquiry. In recent years, these “ensemble” forecasting approaches frequently have been among the top methods used in prediction challenges across a wide range of applications.

Ensembles are a natural choice for noisy, complex, and interdependent systems that evolve over time. In these settings, no one model is likely to be able to capture and predict the full set of complex relationships that drive future observations from a particular system of interest. Instead “specialist” or “component” models can be relied on to capture distinct features or signals from a system and, when combined, represent a nearly complete range of possible outcomes. In this work, we develop and compare a collection of ensemble methods for combining predictive densities. This enables us to quantify the improvement in predictions achieved by using ensemble methods with varying levels of complexity.

To illustrate these ensemble methods, we present time-series forecasts for infectious disease, specifically for influenza in the United States. The international significance of emerging epidemic threats in recent decades has highlighted the importance of understanding and being able to predict infectious disease dynamics. With the revolution in science driven by the promise of “big” and real-time data, there is an increased focus on and hope for using statistics to inform public health policy and decision-making in ways that could mitigate the impact of future outbreaks. Some of the largest public health agencies in the world, including the US Centers for Disease Control and Prevention (CDC) have openly endorsed using models to inform decision making, saying “with models, decision-makers can look to the future with confidence in their ability to respond to outbreaks and public health emergencies” [1].

There is a large literature on prediction methods for influenza. We will give a brief overview of this literature here, and refer the reader to Chretien *et al.* [2] and Nsoesie *et al.* [3] for more comprehensive reviews; additionally, [4] present the results of a recent influenza prediction challenge run by the CDC where many of these models were employed. Infectious disease prediction methods can be broadly grouped into three categories: agent-based models, compartmental models [5–10], and regression-based time series models that may include auto-regressive and seasonal terms [11]. Additionally, these models may use a variety of different data sources and covariates to inform their predictions, including historical values of the disease incidence time series [5–11]; data derived from internet sources such as web searches, wikipedia page views, and twitter [5, 6, 8, 11–14]; and climatological variables [5, 6, 8, 10, 11], among others. These models may generate either point predictions, possibly along with associated predictive intervals, or full predictive distributions.

The ensemble methods that we explore in the present work are designed to combine predictions from multiple models, which could use a variety of different model structures and covariates to generate predictions. Development of the methods presented in this manuscript was motivated by the observation that certain prediction models for infectious disease consistently performed better than other models at certain times of year. We observed in previous research that early in the influenza season, simple models of historical incidence often outperformed more standard time-series prediction models such as a seasonal auto-regressive integrated moving average (SARIMA) model [15]. However, in the middle of the season, the time-series models showed improved accuracy. We set out to determine whether ensemble methods could use this information about past model performance to improve predictions.

A large number of ensemble methods have been developed for a diverse array of tasks including regression, classification, and density estimation. These methods are broadly similar in that they combine results from multiple component models. However, details differ between ensemble methods. We suggest Polikar [16] for a review of ensemble methods; many of these are also discussed in detail in Hastie *et al.* [17].

While there are many different methods for combining models, all ensemble models discussed in this paper use an approach called stacking [18]. In this approach, each of the component models is trained separately in a first stage, and cross-validated measures of performance of those component models are obtained. Then, in a second stage, a stacking model is trained using the cross-validated performance measures to learn how to optimally combine predictive densities from the component models. The specific implementations of stacking that we use obtain the final predictive density as a weighted sum of the component predictive densities, where the weights may depend on covariates. We refer to this approach generally as a “weighted density ensemble” approach to prediction. Several variations on this strategy have been explored in the literature previously [19–21]. However, other ensemble methods for density estimation have also been developed. For example, Rosset and Segal [22] develop a boosting method in which the component models are estimated sequentially, with results from earlier models affecting estimation of later models.

In structured prediction settings such as time series forecasting, ensemble methods may benefit from taking advantage of the data structure. For example, it may be the case that different models offer a better representation of the data at different points in time. A common idea in these settings is to use model weights that change over time. For instance, model weights may vary as a function of how well each model did in recent predictions [23] or by using a more formal graphical structure such as a hidden Markov model to track which component model is most likely to have generated new observations as they arise over time [24, 25]. It is also possible to combine the component models with weights that depend on observed covariates or features [26]. For example, in an ensemble for a user recommendation system, Jahrer *et al.* [27] allowed model weights to depend on a variety of features including the time that a user submitted a rating.

Using component models that generate predictive densities for outcomes of interest, we have implemented a series of ensembles using different methods for choosing the weights for each model. Specifically, we compare three different approaches. The first approach simply takes an equally weighted average of all models. The second approach estimates constant but not necessarily equal weights for each model. The third approach is a novel method for determining model weights based on features of the system at the time predictions are made. The overarching goal of this study is to create a systematic comparison between ensemble methods to study the benefits of increasing complexity in ensemble weighting schemes.

We are aware of two previous articles that developed ensemble methods for infectious disease prediction. Yamana *et al.* [28] and Chakraborty *et al.* [11] both developed model stacking frameworks that are similar to the second approach outlined above using a constant weight for each component model. The present article is differentiated from this previous work in that we explore and compare a range of more flexible ensemble methods where the weights depend on observed features.

This paper presents a novel ensemble method that determines optimal model combinations based on (a) observed data at the time predictions are made and (b) aspects of the predictive distributions obtained from the component models. We refer to models built using this approach as “feature-weighted” ensembles. This approach fuses aspects of different ensemble methods: it uses model stacking [18] and estimates model weights based on features of the system [26] using gradient tree boosting [29].

Using seasonal influenza outbreaks in the US health regions as a case-study, we developed and applied our ensemble models to predict several attributes of the influenza season at each week during the season. By illustrating the utility of these approaches to ensemble forecasting in a setting with complex population dynamics, this work highlights the importance of continued innovation in ensemble methodology.

## Methods

This paper presents a comparison of methods for determining weights for weighted density ensembles, applied to forecasting specific features of influenza seasons in the US. First, we present a description of the influenza data we use in our application and the prediction targets. Next, we discuss the three component models utilized by the ensemble framework. We then turn to the ensemble framework itself, describing the different ensemble model specifications used.

### Data and prediction targets

**Fig 1:**
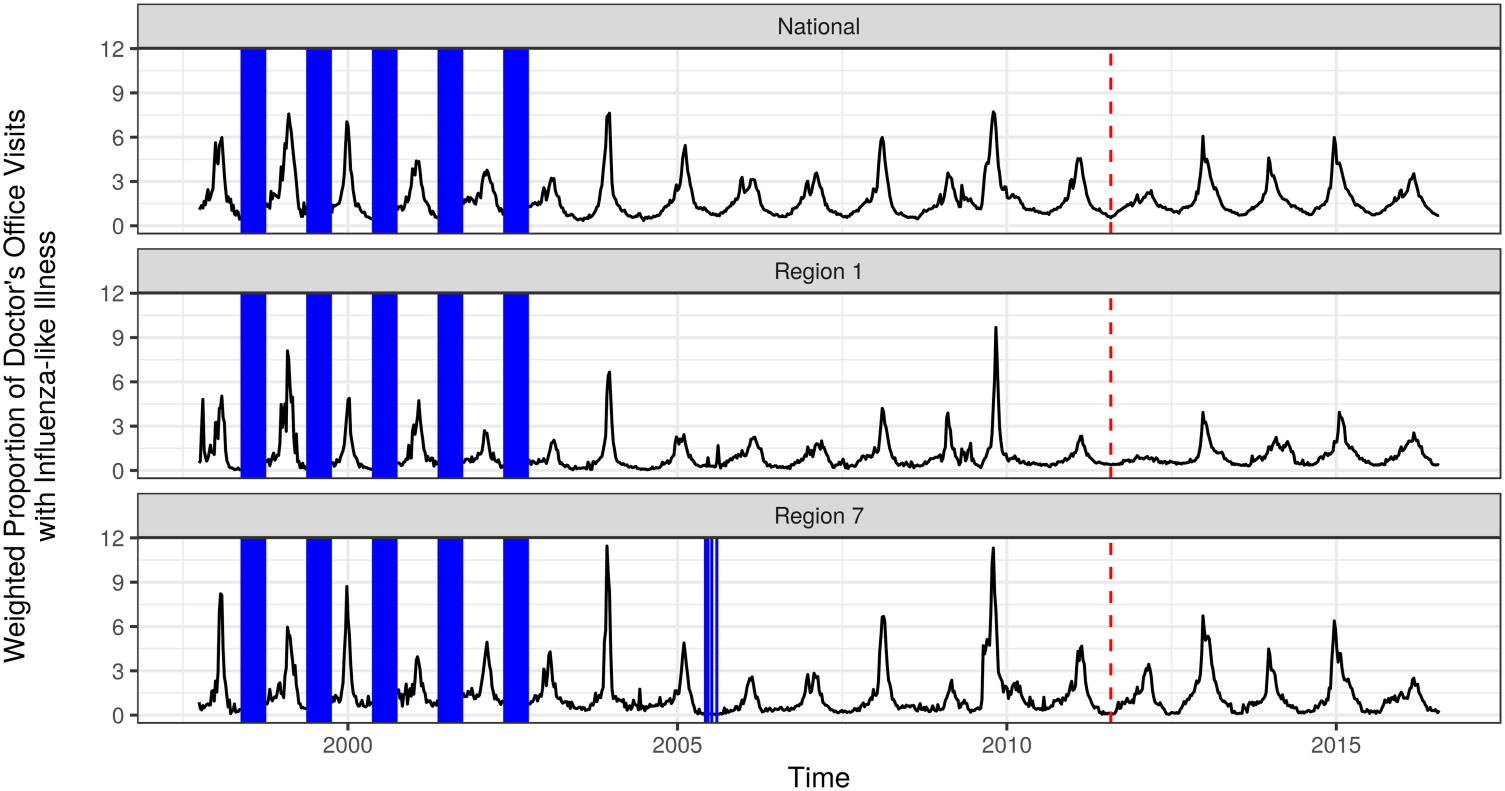
Plot of influenza data. The full data include observations aggregated to the national level and for ten smaller regions. Here we plot only the data at the national level and in two of the smaller regions; data for the other regions are qualitatively similar. Missing data are indicated with vertical blue bars. The vertical red dashed lines indicate the cutoff time between the training and testing phases; five seasons of data were held out for testing.

We obtained publicly available data on seasonal influenza activity in the United States between 1997 and 2016 from the US Centers for Disease Control and Prevention (CDC) (Fig 1). For each of the 10 Health and Human Services regions in the country in addition to the nation as a whole, the CDC calculates and publishes each week a measure called the weighted influenza-like illness (wILI) index. The wILI for a particular region is calculated as the average proportion of doctor visits with influenza-like illness for each state in the region, weighted by state population. During the CDC-defined influenza season (between Morbidity and Mortality Weekly Report week 40 of one year and 20 of the next year), the CDC publishes updated influenza data on a weekly basis. This includes “current” wILI data from two weeks prior to the reporting date, as well as updates to previously reported numbers as new data becomes available. For this analysis, we use only the final reported wILI measures to train and predict from our models. In the early seasons, data were not recorded during the off-season. Additionally, there were 52 observations in which the reported wILI was zero; these generally occurred near the off-season in early years, and occurred in weeks when only small numbers of health care providers submitted reports to the CDC. We treated these reported zeros as missing data throughout the analysis.

The CDC defines the influenza season onset as the first of three consecutive weeks of the season for which wILI is greater than or equal to a threshold that is specific to the region and season. This threshold is the mean percent of patient visits where the patient had ILI during low incidence weeks for that region in the past three seasons, plus two standard deviations [30]. The CDC provides historical threshold values for each region going back to the 2007/2008 season [31]. Additionally, we define two other metrics specific to a region-season. The peak incidence is the maximum observed wILI measured in a season. The peak week is the week at which the maximum wILI for the season is observed.

Each predictive distribution was represented by probabilities assigned to bins associated with different possible outcomes. For onset week, the bins are represented by integer values for each possible season week plus a bin for “no onset”. For peak week, the bins are represented by integer values for each possible season week. For peak incidence, the bins capture incidence rounded to a single decimal place, with a single bin to capture all incidence over 12.95. Formally, the incidence bins are as follows: [0, 0.05), [0.05, 0.15), …, [12.85, 12.95), [12.95, 100]. These bins were used in the 2016-2017 influenza prediction contest run by the CDC [32].

We measure the accuracy of predictive distributions using the log score. The log score is a proper scoring rule [33], calculated in our setting as the natural log of the probability assigned to the bin containing the true observation. Proper scoring rules are preferred for measuring the quality of predictive distributions because the expected score is optimized by the true probabilty distribution. We note that for peak week, in some region-seasons the same peak incidence was achieved in multiple weeks (after rounding to one decimal place). In those cases, we calculated the log score as the log of the sum of the probabilities assigned to those weeks; this is consistent with scoring procedures used in the 2016-2017 flu prediction contest run by the CDC [32]. However, the log score is not directly comparable with the score used by the CDC in the prediction contest. The CDC calculates the score of a prediction as the log of the combined probability assigned to several bins surrounding the realized outcome; this has some benefits, but has the disadvantage that it is not a proper score. We have opted to use the log score in this work because it is a proper score.

### Component models

We used three component models to generate probabilistic predictions of the three prediction targets. The first model was a seasonal average model that utilized kernel density estimation (KDE) to estimate a predictive distribution for each target. The second model utilized kernel conditional density estimation (KCDE) and copulas to create a joint predictive distribution for incidence in all remaining weeks of the season, conditional on recent observations of incidence [15]. By calculating appropriate integrals of this joint distribution, we constructed predictive distributions for each of the seasonal targets. The third model used a standard seasonal auto-regressive integrated moving average (SARIMA) implementation. All models were fit independently on data within each region.

#### Kernel density estimation (KDE)

The simplest of the component models uses kernel density estimation [34] to estimate a distribution for each target based on observed values of that target in previous seasons within the region of interest. We used Gaussian kernels and the default settings from the density function in the stats package for R [35] to estimate the bandwidth parameter. For the peak incidence target, we fit to log-transformed observations of historical peak incidence. For the onset week prediction target, we estimated the probability of no onset as the proportion of region-seasons in all regions in the training phase where no week in the season met the criteria for being a season onset.

To create an empirical predictive distribution of size *N* from a KDE fit based on a data vector **y**_1:*K*_ (for example, this might be the vector of peak week values from the *K* training seasons), we first drew *N* samples with replacement from **y**_1:*K*_, yielding a new vector ${\tilde{y}}_{1:N}$. We then drew a single psuedo-random deviate from each of *N* truncated Gaussian distributions centered at ${\tilde{y}}_{1:N}$ with the bandwidth estimated by the KDE algorithm. The Gaussians we sampled from were truncated at the lower and upper bounds of possible values for the given prediction target. Finally, we discretized the sampled values to the target-specific bins. These sampled points then make up the empirical predictive distribution from a KDE model. We set the sample size to *N* = 10^5^. In theory, this model assigns non-zero probability to every possible outcome; however, in a few cases the empirical predictive distribution resulting from this Monte Carlo sampling approach assigned probability zero to some of the bins.

It is important to note that the predictions from this model do not change as new data are observed over the course of the season.

#### Kernel conditional density estimation (KCDE)

We used kernel conditional density estimation and copulas to estimate a joint predictive distribution for flu incidence in each future week of the season, and then calculated predictive distributions for each target from that joint distribution [15]. In our implementation, we first used KCDE to obtain separate predictive densities for flu incidence in each future week of the season. Each of these predictive densities gives a conditional distribution for incidence at one future time point given recent observations of incidence and the current week of the season. KCDE can be viewed as a distribution-based analogue of nearest-neighbors regression. We then used a copula to model dependence among those individual predicitive densities, thereby obtaining a joint predicitive density, or a distribution of incidence trajectories in all future weeks.

To predict seasonal quantities (onset, peak timing, and peak incidence), we simulate *N* = 10^5^ trajectories of disease incidence from this joint predictive distribution. For each simulated incidence trajectory, we compute the onset week, peak week, and peak incidence. We then aggregate these values to create predictive distributions for each target. This procedure for obtaining predictive distributions for the targets of interest can be formally justified as an appropriate Monte Carlo integral of the joint predictive distribution for disease incidence in future weeks (see [15] for details).

#### Seasonal auto-regressive integrated moving average (SARIMA)

We fit seasonal ARIMA models [36] to wILI observations transformed to be on the natural log scale. We manually performed first-order seasonal differencing and used the stepwise procedure from the auto.arima function in the forecast package [37] for R to select the specification of the auto-regressive and moving average terms.

Similar to KCDE, forecasts were obtained by sampling *N* = 10^5^ trajectories of wILI values over the rest of the season (using the simulate.Arima function from the forecast package), and predictive distributions of the targets were computed from these sampled trajectories as described above.

#### Component model training

We used data from 14 seasons (1997/1998 through 2010/2011) to train the models. Data from five seasons (2011/2012 through 2015/2016) were held out when fitting the models and used exclusively in the testing phase. To avoid overfitting our models, we made predictions for the test phase only once [17].

Estimation of the ensemble models (discussed in the next subsection) requires cross-validated measures of performance of each of the component models in order to accurately gauge their relative performance. For each region, we estimated the parameters of each component model 15 times: 14 fits were obtained excluding one training season at a time, and another fit used all of the training data. For each fit obtained leaving one season out, we generated a set of three predictive distributions (one for each of the prediction targets) at each week in the held-out season. We were not able to generate predictions from the SARIMA and KCDE models for some seasons in the training phase because those models used lagged observations from previous seasons that were missing in our data set. The component model fits based on all of the training data were used to generate predictions for the test phase.

### Ensemble models

All of the ensemble models we consider in this article work by averaging predictions from the component models to obtain the ensemble prediction. Additionally, these methods are stacked model ensembles because they use leave-one-season-out predictions from the independently estimated component models as inputs to estimate the model weights [18]. We begin our discussion of ensemble methods with a general overview, introducing a common set of notation and giving a broad outline of the ensemble models we will use in this article. We then describe our proposed weighted density ensemble model specifications in more detail.

#### Overview of ensemble models

A single set of notation can be used to describe all of the ensemble frameworks implemented here. Let $f_{m}(y_{t}|x_{t}^{\left(m\right)})$ denote the predictive density from component model *m* for the value of the scalar random variable *Y*_*t*_ conditional on observed variables $x_{t}^{\left(m\right)}$. Observations of disease incidence are reported weekly in our data set, so *t* indexes the week of the season. The variable *Y*_*t*_ could for example represent the peak incidence for a given season and region; in our application to predicting seasonal quantities, the same outcome *y*_*t*_ will be realized for all weeks within a given season. In the context of time series predictions, the covariate vector $x_{t}^{\left(m\right)}$ may include time-varying covariates such as the week at which the prediction is made or lagged incidence. The superscript ^(*m*)^ reflects the fact that each component model may use a different set of covariates.

The combined predictive density *f*(*y*_*t*_|**x**_*t*_) for a particular target can be written as

$$\begin{array}{c} f\left(y_{t}|x_{t}\right)=\sum_{m=1}^{M}\pi_{m}\left(x_{t}\right)f_{m}\left(y_{t}|x_{t}^{\left(m\right)}\right). \end{array}$$ (1)

**Fig 2:**
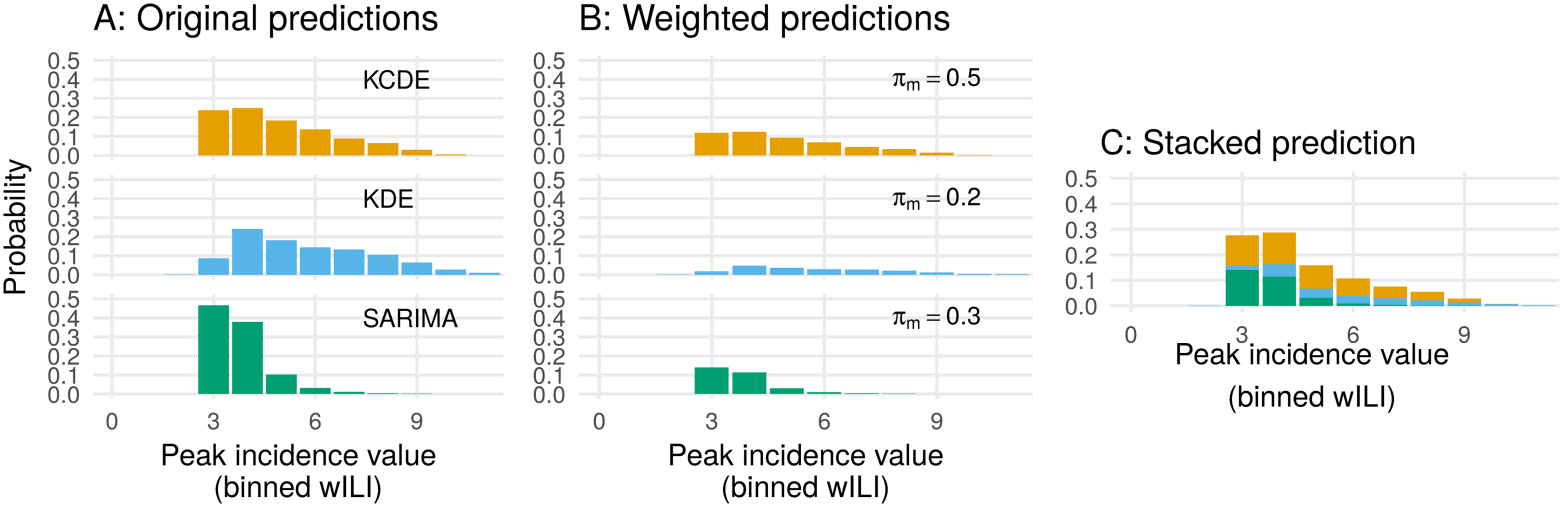
Conceptual diagram of how the stacking models operate on probabilistic predictive distributions. The distributions illustrated here have density bins of 1 wILI unit, which differs from those used in the manuscript for illustrative purposes only. Panel A shows the predictive distributions from three component models. Panel B shows scaled versions of the distributions from A, after being multiplied by model weights. In Panel C, the scaled distributions are literally stacked to create the final ensemble predictive distribution.

In Eq (1) the *π*_*m*_ are the model weights, which are allowed to vary as a function of observed features in **x**_*t*_. We define **x**_*t*_ to be a vector of all observed quantities that are used by any of the component models or in calculating the model weights. In order to guarantee that *f*(*y*_*t*_|**x**_*t*_) is a probability distribution we require that $\sum_{m=1}^{M}\pi_{m}(x_{t})=1$ for all **x**_*t*_. Fig 2 illustrates the concept of stacking the predictive densities for each component model.

In the following subsection, we propose a framework for estimating *feature-dependent weights* for a stacked ensemble model. By *feature-dependent* we mean that the weights associated with different component models are driven by observed features or covariates. Although we illustrate the method in the context of time-series predictions, the method could be used in any setting where we wish to combine distribution estimates from multiple models. Features could include observed data from the system being predicted (such as recent wILI measurements or the time of year at which predictions are being made), observed data from outside the system (for example, recent weather observations), or features of the predictions themselves (e.g. summaries of the predictive distributions from the component models, such as a measure of spread in the distribution, or the time until a predicted peak). Based on exploration of training phase data and *a priori* knowledge of the disease system, we chose three features of the system to illustrate the proposed “feature-weighting” methodology: week of season, component model uncertainty (defined as the minimum number of predictive distribution bins required to cover 90% probability), and wILI measurement at the time of prediction. These features were chosen prior to and not changed after implementing test-phase predictions.

We used four distinct methodologies to define weights to use for the stacking models:

1. Equal Weights (**EW**): *π*_*m*_(**x**_*t*_) = 1/*M*. In this scenario, each model contributes the same weight for each target and for all values of **x**_*t*_.
2. Constant model weights via degenerate EM (**CW**): *π*_*m*_(**x**_*t*_) = *c*_*m*_, a constant where $\sum_{m=1}^{M}c_{m}=1$ but the constants are not necessarily the same for each model. These weights are estimated using the degenerate estimation-maximization algorithm [38]. A separate set of weights is estimated for each region and prediction target.
3. Feature-weighted (**FW-wu**): *π*_*m*_(**x**_*t*_) depends on features including week of the season and model uncertainty for the KCDE and SARIMA models. A separate set of weighting functions is estimated for each region and prediction target.
4. Feature-weighted with regularization: *π*_*m*_(**x**_*t*_) depends on features, but with regularization discouraging the weights from taking extreme values or from varying too quickly as a function of **x**_*t*_. A separate set of weighting functions is estimated for each region and prediction target. We fit three variations on this ensemble model, using different sets of features:
  - (**FW-reg-w**) week of the season;
  - (**FW-reg-wu**) week of the season and model uncertainty for the KCDE and SARIMA models;
  - (**FW-reg-wui**) week of the season, model uncertainty for the KCDE and SARIMA models, and incidence (wILI) in the most recent week.

All in all, this leads to 6 ensemble models, summarized in Table 1. The first three of these models (**EW**, **CW**, and **FW-wu**) can be viewed as variations on **FW-reg-wu** if we vary the amount and type of regularization imposed on the **FW-reg-wu** model. Thus, comparisons among these four models will enable us to explore the benefits of allowing the model weights to depend on covariates while imposing an appropriate amount of rigidity on the model weight functions *π*_*m*_(**x**_*t*_). We will discuss the regularization strategies used in **FW-reg-wu** further in the next subsection. Meanwhile, comparisons among the **FW-reg-w**, **FW-reg-wu**, and **FW-reg-wui** models will allow us to explore the relative contributions to predictive performance that can be achieved by allowing the model weights to depend on different features.

**Table 1:** Summary of ensemble methods and what the model weights depend on.

Model	Component Model Weights Vary with…					
	Region	Prediction Target	Week of Season	SARIMA Uncertainty	KCDE Uncertainty	Current wILI
EW						
CW	X	X				
FW	X	X	X	X	X	
FW-reg-w	X	X	X			
FW-reg-wu	X	X	X	X	X	
FW-reg-wui	X	X	X	X	X	X

As discussed above, leave-one-season-out prediction results from the three component models are inputs to the ensemble estimation routines. During ensemble estimation, we dropped any training set time points for which cross-validated predictions from all three component models were not available. After the training phase, each of the six ensemble models, along with the three component models, are used to generate predictions in every season-week of each of the five testing seasons, assuming perfect reporting. These predictions are then used to evaluate the prospective predictive performance of each of the ensemble methods. In total, we evaluate 9 models in 11 regions over 5 years and 3 targets of interest.

### Feature-weighted stacking framework

In this section we introduce the particular specification of the parameter weight functions *π*_*m*_(**x**_*t*_) that we use for the **FW-wu**, **FW-reg-w**, **FW-reg-wu**, and **FW-reg-wui** models and discuss estimation.

In order to ensure that the the *π*_*m*_ are non-negative and sum to 1 for all values of **x**_*t*_, we parameterize them in terms of the softmax transformation of real-valued latent functions *ρ*_*m*_:

$$\begin{array}{c} \pi_{m}\left(x_{t}\right)=\frac{\exp\{\rho_{m}\left(x_{t}\right)\}}{\sum_{m^{\prime }=1}^{M}\exp\left\{\rho_{m^{\prime }}\left(x_{t}\right)\right\}}. \end{array}$$ (2)

For a pair of models *l*, *m* ∈ {1, …, *M*}, *ρ*_*l*_(**x**_*t*_) > *ρ*_*m*_(**x**_*t*_) indicates that model *l* has more weight than model *m* for predictions at the given value of **x**_*t*_. The functions *ρ*_*m*_(**x**_*t*_) could be parameterized and estimated using many different techniques, such as a linear specification in the features, splines, or so on. We chose to estimate the functions *ρ*_*m*_(**x**) using gradient tree boosting.

Gradient tree boosting uses a forward stagewise additive modeling algorithm to iteratively and incrementally construct a series of regression trees that, when added together, create a function designed to minimize a given loss function. In our application, the algorithm builds up the *ρ*_*m*_(**x**_*t*_) that minimize the negative log-score of the stacked predictions *f*(*y*_*t*_|**x**_*t*_) across all times *t*:

$$\begin{array}{cc} L\{\rho \left(x_{t}\right)\} & =-\sum_{t}\log\left\{f\left(y_{t}|x_{t}\right)\right\}\\ & =-\sum_{t}\log[\sum_{m=1}^{M}\frac{\exp\{\rho_{m}\left(x_{t}\right)\}}{\sum_{m^{\prime }=1}^{M}\exp\left\{\rho_{m^{\prime }}\left(x_{t}\right)\right\}}f_{m}\left(y_{t}|x_{t}^{\left(m\right)}\right)], \end{array}$$ (3)

where $f_{m}(y_{t}|x_{t}^{\left(m\right)})$ is the cross-validated predictive density from the *m*th model evaluated at the realized outcome *y*_*t*_.

Specifically, we define a single tree as

$$\begin{array}{c} T\left(x_{t};\theta \right)=\sum_{j=1}^{J}\gamma_{j}I_{R_{j}(\psi )}\left(x_{t}\right), \end{array}$$ (4)

where the *R_j_*(***ψ***) are a set of disjoint regions that comprise a partition of the space X of feature values **x**_*t*_, and *I* is the indicator function taking the value 1 if **x**_*t*_ ∈ *R_j_*(***ψ***) and 0 otherwise. The parameters ***θ*** = (***ψ***, ***γ***) for the tree are the split points ***ψ*** partitioning X into the regions *R_j_*(***ψ***) and the regression constants ***γ*** associated with each region. The function *ρ*_*m*_(**x**_*t*_) is obtained as the sum of *B* trees:

$$\begin{array}{c} \rho_{m}\left(x_{t};\Theta_{m}\right)=\sum_{b=1}^{B}T\left(x_{t};\theta_{m,b}\right). \end{array}$$ (5)

In each iteration *b* of the boosting process, we estimate *M* new regression trees, one for each component model. These trees are estimated so as to minimize a local approximation to the loss function around the weight functions that were obtained after the previous boosting iteration. Our approach builds on the xgb.train function in the xgboost package for R to perform this estimation [39]. The functionality in that package assumes that the loss function is convex, and optimizes a quadratic approximation to the loss in each boosting iteration. The loss function in Eq (3) is not guaranteed to be convex, so a direct application of this optimization method fails in our setting. We have modified the implementation in the xgboost package to use a gradient descent step in cases where the loss is locally nonconvex (concave or indeterminate).

Gradient tree boosting is appealing as a method for estimating the functions *ρ*_*m*_ because it offers a great deal of flexibility in how the weights can vary as a function of the features **x**_*t*_. On the other hand, this flexibility can lead to overfitting the training data. In order to limit the chances of overfitting, we have explored the use of three regularization parameters:

1. The number of boosting iterations *B*. As *B* increases, more extreme weights (close to 0 or 1) and more rapid changes in the weights as **x** varies are possible.
2. An *L*_1_ penalty on the number of tree leaves, *J*. A large penalty encourages the regression trees to have fewer leaves, so that there is less flexibility for the model weights to vary as a function of **x**_*t*_.
3. An *L*_1_ penalty on the regression constants *γ*_*j*_. A large penalty encourages these constants to be small, so that the overall model weights change less in each boosting iteration.

We selected values for these regularization parameters using a grid search optimizing leave-one-season-out cross-validated model performance.

### Software and code

We used R version 3.2.2 (2015-08-14) for all analyses [35]. All data and code used for this analysis is freely available in an R package online at https://github.com/reichlab/adaptively-weighted-ensemble and may be installed in R directly. Predictions generated in real-time with early development versions of this model during the 2016/2017 influenza season may be viewed at https://reichlab.io/flusight/. To maximize reproducibility of our work, we have set seeds prior to running code that relies on stochastic simulations using the rstream package [40]. Additionally, the manuscript itself was dynamically generated using RMarkdown.

## Results

To evaluate overall model performance, we computed log scores for all predictions made by each model across all regions and test phase seasons. Predictions made before the season peak (for predictions of peak incidence or peak timing) or before the season onset (for predictions of season onset timing) are the most relevant to decision makers using the predictions as inputs to set public policy. We therefore focus our comparison of model performance on results for predictions made before the target event occurred within each of the test phase seasons. Plots of the full predictive distributions at the national level from the **FW-reg-w** ensemble are presented in S1, S2 and S3 Figs.

As discussed in the methods section, our test set contained predictions from each model for 3 targets over 5 seasons in 11 spatial units. To ensure that seasons with later onsets or later peaks would not count more heavily than seasons with earlier onsets and peaks, and to simplify the analysis in the presence of serial autocorrelation in model performance over consecutive weeks, we summarized model performance within each season by the mean log score for all predictions made before the peak or onset week (as appropriate for the prediction target). This led to 165 observations of model performance for each model, corresponding to the unique combinations of prediction target, season, and spatial unit.

### Feature-weighted ensemble model weights reflect trends in component model log scores

**Fig 3:**
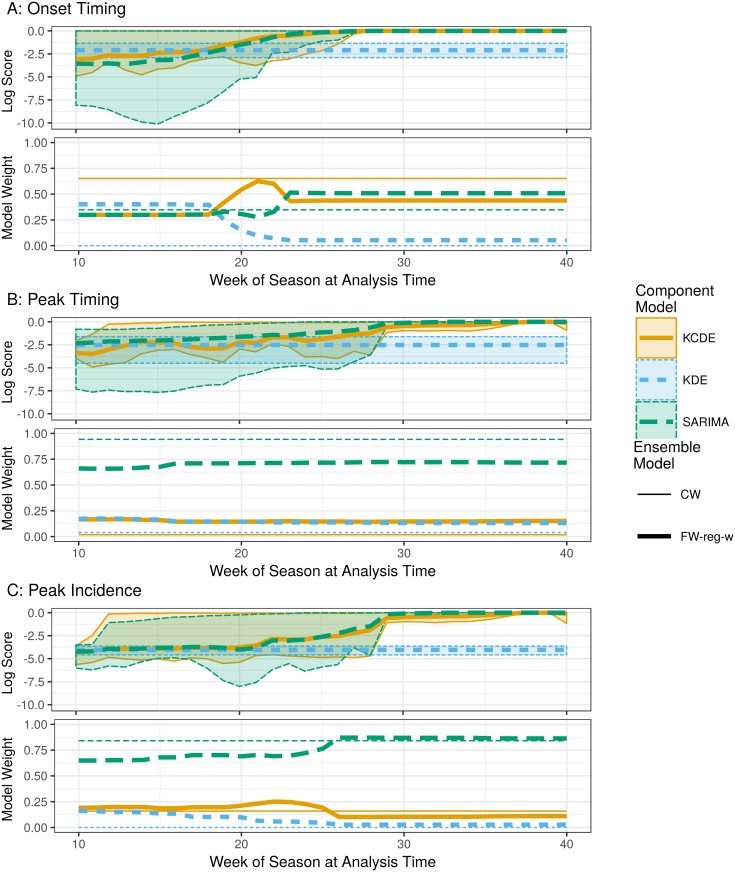
Example of component model weights from the CW and FW-reg-w models. Model weights are shown for predictions of onset timing (panel A), peak timing (panel B), and peak incidence (panel C) at the national level. The upper plot within each panel shows mean, minimum, and maximum log scores achieved by each component model for predictions of the given prediction target at the national level in each week of the season, summarizing across all seasons in the training phase when all three component models produced predictions. The lower plot within each panel shows model weights from the textbfCW and textbfFW-reg-w ensemble methods at each week in the season.

Fig 3 displays variation in leave-one-season-out log scores from the three component models over the course of the training phase seasons, along with the corresponding model weight estimates from the **CW** and **FW-reg-w** models. Performance of the **SARIMA** and **KCDE** models is similar, with mean log scores from those models starting out near or slightly below the mean performance of **KDE**, but with performance improving as more data become available. Near the beginning of some seasons, predictions from the **SARIMA** model are quite a bit worse than predictions from the other two component models. S4 Fig illustrates that these patterns are consistent across the other regions. S5 Fig shows that performance of the component models also varies with the model’s uncertainty as measured by the number of bins required to cover 90% in the predictive distribution, and S6 Fig shows that performance varies with the observed wILI in the week when predictions are made.

The model weights assigned by the feature weighted ensemble models generally track these trends in relative model performance (Fig 3, S7 Fig). For all three targets, at the national level the weight assigned to the **SARIMA** model increases and the weight assigned to **KDE** decreases as the season progresses. However, the magnitude of shifts in model weights as the weighting features vary is different for the three prediction targets.

### Best models have similar aggregate performance

**Fig 4:**
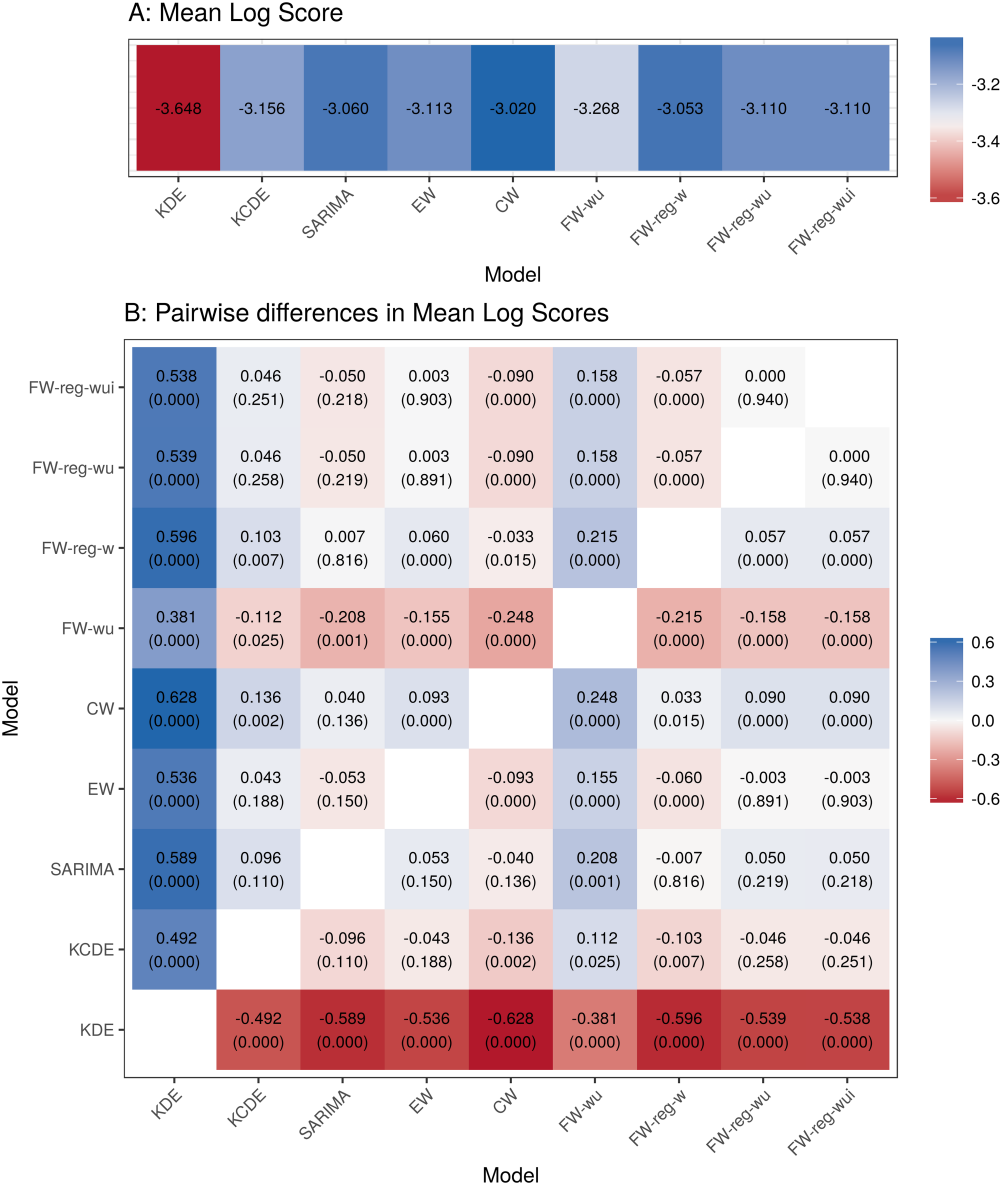
Permutation test results for pairwise comparisons of the mean log scores for each method. For each combination of 3 prediction targets, 11 regions, and 5 test phase seasons, we calculated the mean log score for all predictions made by each method in weeks before the event being predicted occurred. Panel A presents the overall mean of these values for each method; higher mean log scores indicate better performance. Panel B displays the difference in mean log scores for each pair of models. Positive values indicate that the model on the vertical axis outperformed the model on the horizontal axis on average. A permutation test was used to obtain approximate p-values for these differences (see S1 Text for details). For reference, a Bonferroni correction at a familywise significance level of 0.05 for all pairwise comparisons leads to a significance cutoff of approximately 0.0014.

Aggregating across all combinations of prediction target, region, and season in the test phase, the best component models and the best ensemble models had similar performance (Fig 4). The **CW** ensemble had the highest average log scores across all three prediction targets, but a permutation test (described in S1 Text) was unable to distinguish its performance from the **KCDE**, **SARIMA**, or **FW-reg-w** models. However, these four methods all outperformed the **KDE** model in terms of mean log scores by a wide margin, as well as the **EW** and **FW-wu**, **FW-reg-wu**, and **FW-reg-wui** ensembles by narrower margins. These general trends in model performance were similar for each of the three prediction targets individually; for example, S8 Fig demonstrates that average performance of the **FW-reg-w** and **SARIMA** models is similar for all three prediction targets.

As noted above, our test set included only 5 seasons, and the effective sample size for model comparison is smaller than the 165 combinations of prediction target, region, and test phase season due to correlations in predictive performance across regions and seasons. This may have contributed to our inability to detect statistically significant differences between the best models, and may limit the generalizability of these results; we will return to this point in the discussion.

### Ensembles show stable performance for early-season predictions

Although the aggregate performance of these models is quite similar, some differences between the methods begin to emerge when we examine performance in more detail. Predictions that are used in setting public policy must be of consistent quality across all regions and seasons. We observed that the component models showed more variability and lower worst-case performance than the ensemble methods. The discussion in this subsection presents results of an exploratory analysis of the results, and all p-values are from post-hoc hypothesis tests.

**Fig 5:**
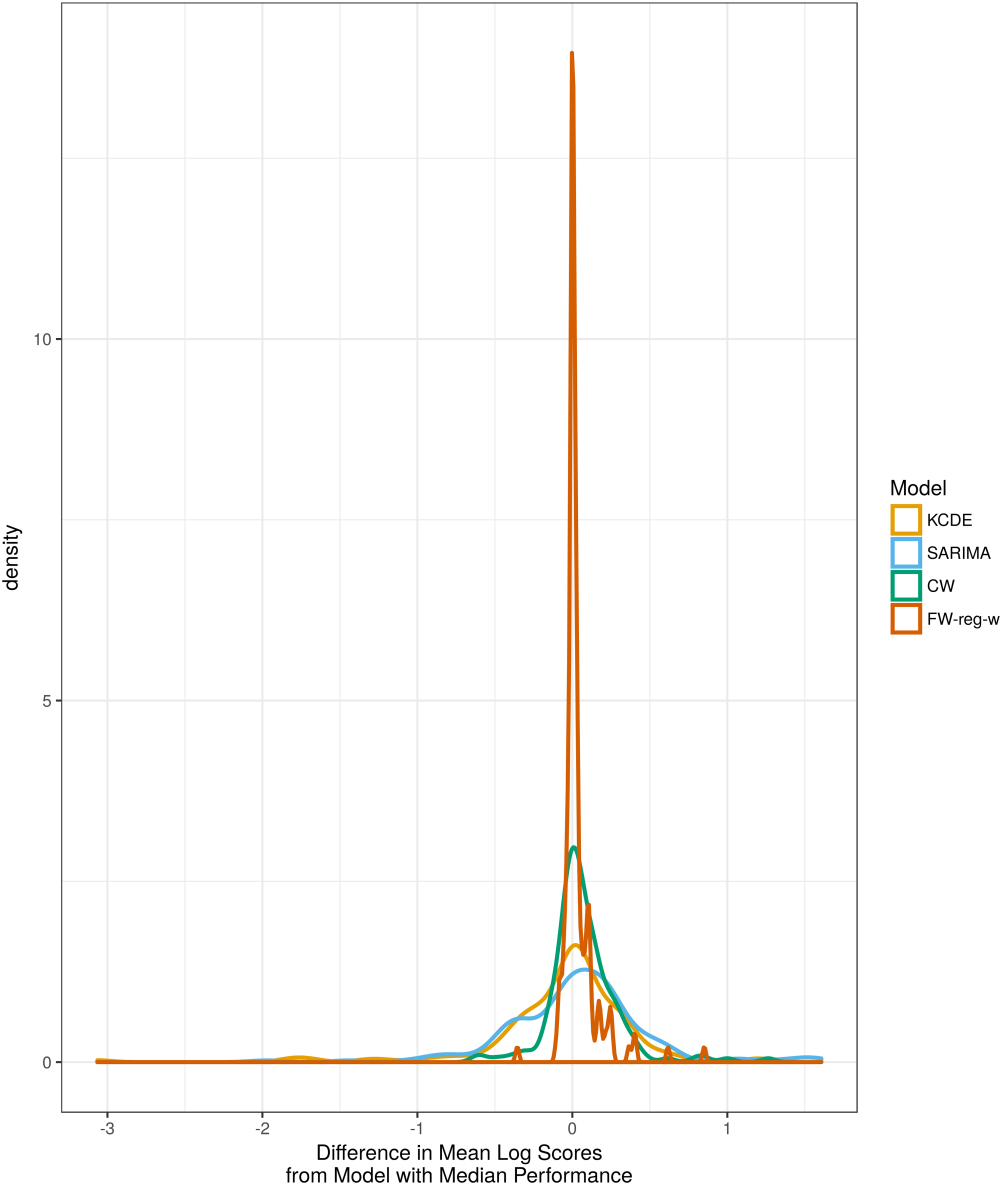
Density plots summarizing differences in mean log scores for each method relative to the median model. We calculate the difference in log scores for a given method and the method with median performance for each combination of prediction target, region, and test phase season; each density curve summarizes results across all 165 combinations of 3 prediction targets, 11 regions, and 5 test phase seasons. Positive values indicate better performance than the median model. For legibility, we only show results for the two component models with best mean performance (KCDE and SARIMA) and for the two ensemble models with best mean performance (CW and FW-reg-w).

To examine consistency of predictive performance, for each combination of prediction target, region, and test phase season we calculated the difference in mean log scores between each method and the method with median performance for that target, region, and season. This measure of model performance relative to the median can be compared across prediction targets, regions, and seasons that may be predicted with varying levels of difficulty. Fig 5 displays these differences in performance relative to the median for just the **KCDE**, **SARIMA**, **CW**, and **FW-reg-w** methods. This comparison demonstrates that while these methods all had similar average performance, the **CW** and **FW-reg-w** ensemble methods had more consistent performance than the component models did, as is observed by the heavier distributional tails below zero on the horizontal axis.

**Fig 6:**
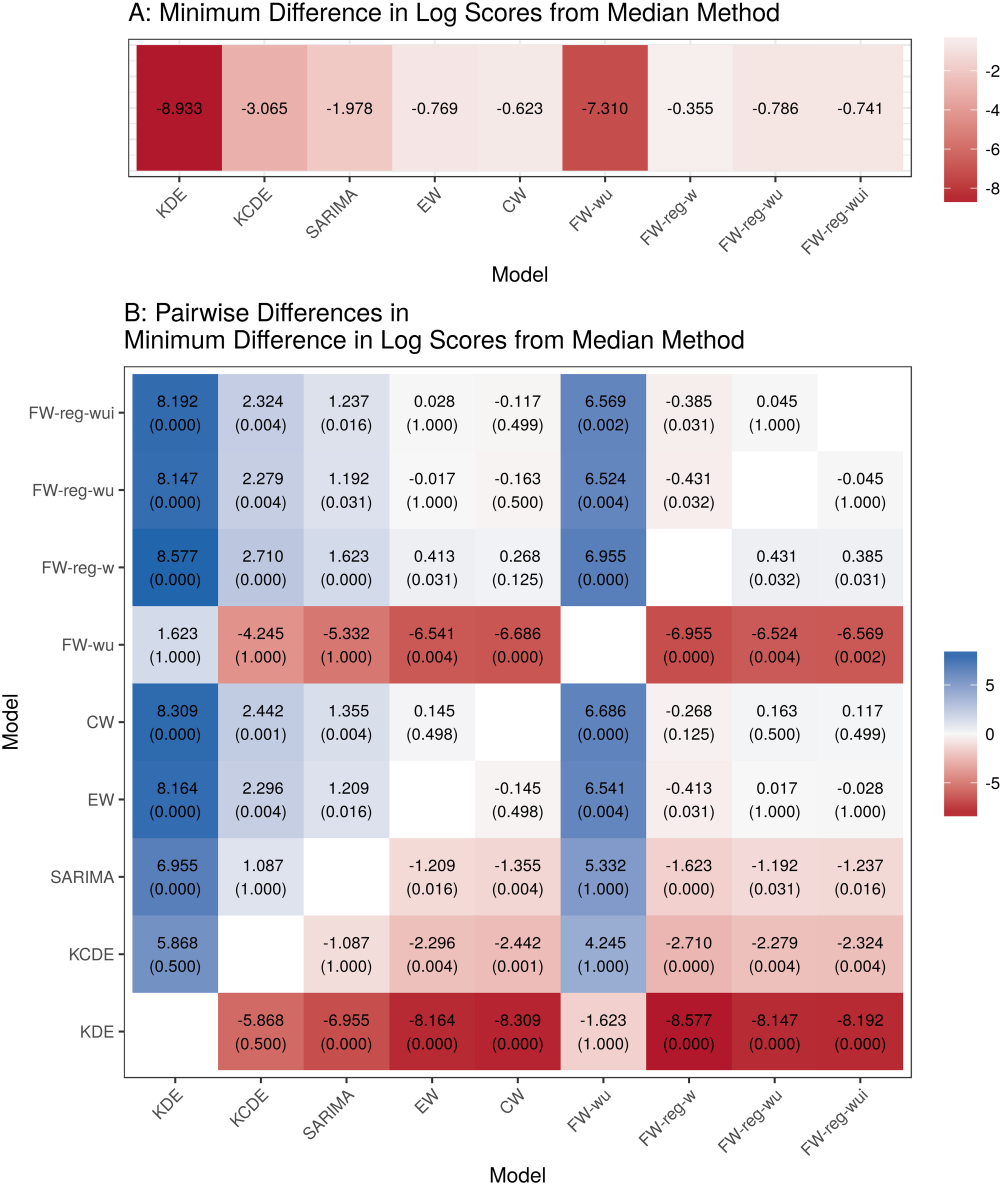
Permutation test results for comparisons of the minimum difference in mean log scores relative to the median for each pair of methods. For each combination of 3 prediction targets, 11 regions, and 5 test phase seasons, we calculated the difference in mean log scores between each method and the method with median performance for that target, region, and season. Panel A presents the minimum difference from the median model for each method across all combinations of target, region, and season. Larger values of this quantity indicate that the given model has better worst-case performance. Panel B displays the difference in this measure of worst-case performance for each pair of models. Positive values indicate that the model on the vertical axis had better worst-case performance than the model on the horizontal axis. A permutation test was used to obtain approximate p-values for these differences (see S1 Text for details). For reference, a Bonferroni correction at a familywise significance level of 0.05 for all pairwise comparisons leads to a significance cutoff of approximately 0.0014.

We can quantify this observation by comparing the minimum performance relative to the median across all prediction targets, regions, and seasons for each method (Fig 6). This comparison reveals that the **FW-reg-w** ensemble had better worst-case performance than all of the component models, and the **CW** ensemble had better worst-case performance than the **KDE** and **KCDE** component models. These differences were both statistically and practically significant. The differences between the ensemble and component models become more marked if we use the 10th percentile of performance differences relative to the median as a more stable measure of the lower tail of this distribution than the minimum (S9 Fig). Additionally, the **FW-reg-w** model had a higher 10th percentile difference in performance from the median model than all other methods. Across all three prediction targets and all test phase seasons, the **FW-reg-w** ensemble had the most consistent performance of all methods we considered (S10 Fig).

### Regularization improves feature-weighted ensemble models

The regularization of feature-weighted ensembles improved early-season prediction accuracy. A comparison of the **FW-wu** and **FW-reg-wu** models shows improvements in both mean performance and worst-case performance when regularization was used to create smoother functions of model weights as a function of season week and model uncertainty (Figs 4 and 6).

## Discussion

In this work we have examined the potential for ensemble methods to improve infectious disease predictions. We explored a nested series of ensemble methods, focusing on methods that computed weighted averages of predictive distributions for seasonal targets of public health interest, such as the peak intensity of the outbreak and the timing of both season onset and peak. The methods we examined ranged from using equal model weights to more complex schemes with weights that varied as functions of multiple covariates. The best of these ensemble methods achieved overall performance that was about as good as the best of the individual component models, with increased stability in model performance across different regions and seasons.

Increased stability in predictive accuracy can provide decision makers with more confidence when using predictions as inputs to set policy. For example, if a single model does well in most seasons but occasionally fails badly, planning decisions may be negatively impacted in those failing years. This may be particularly important in a public health setting where the events that are most important to get right are those relatively rare cases when incidence is much larger than usual or the season timing is earlier or later than usual. This reduction in variability of model performance achieved by ensemble methods is therefore important for ensuring that our predictions are reliable under a variety of conditions.

Among the different ensemble specifications we considered, the **CW** and **FW-reg-w** models had slightly better average performance during the test phase than the three other ensemble methods that included some form of regularization on the model weighting functions, and much better performance than an ensemble with unregularized weighting function. The **FW-reg-wui** and **FW-reg-wu** ensembles did not outperform the simpler **FW-reg-w** ensemble, indicating that including model uncertainty and recent observations of disease incidence did not add much more information about relative model performance than was available from the week of the season in which predictions were generated. Analysis of worst-case performance suggests that the **FW-reg-w** ensemble had more stable performance across different regions and seasons than the other ensemble specifications. However, whether or not this difference was statistically significant depended on the measure of worst-case performance used. Overall, the **FW-reg-w** method had good average and worst-case performance across all test phase seasons and prediction targets; the **CW** ensemble had similar average performance, but its worst-case performance was not as good as that of the **FW-reg-w** method.

All hypothesis tests we conducted related to worst-case performance were post-hoc tests conducted after an exploratory analysis of relative model performance, and these results should be confirmed in future studies. Additionally, the permutation test we used accounts for serial autocorrelation in model performance within a region-season, but does not account for correlation across region or seasons; thus the p-values discussed throughout this work should be regarded as only approximate indicators of statistical significance.

The feature-weighted ensemble models presented in this article use a novel scheme to estimate feature-dependent model weights that sum to 1 and are therefore suitable for use in combining predictive distributions. This general method could be applied to combine distribution estimates in any context, and is not limited to time-series or infectious disease applications. Furthermore, comparing an implementation of the feature-weighting that smoothed the model weights to one that did not showed consistent improvements in model performance. This result suggests that future work on feature-weighted ensemble implementations should consider regularized estimation.

Infectious disease predictions are only useful to public health officials if they are communicated effectively in real time. Predictions from an early version of the **FW-reg-w** model were updated weekly during the 2016/2017 influenza season and disseminated through an interactive website at https://reichlab.io/flusight/. While we have successfully deployed the methods discussed here in a real-time setting, in this article we have ignored the important issue of reporting delays that occur with real-time data. All models were trained using the finalized value of the incidence measure, and these finalized values were used to make the cross-validated predictions that were inputs to the ensemble estimation as well as the predictions for the test set evaluation. Some component models may be more or less sensitive to reporting delays than other models, and this could lead to inappropriate estimates for the ensemble weighting functions if finalized data were used for the cross-validated predictions but the methods were then used in real time. Ideally, the cross-validated model log scores used to estimate the ensemble weighting functions should be obtained using the same sort of “non-finalized” data that the models will encounter when making real-time predictions.

A central challenge of working with infectious disease data sets is the limited number of years of data available for model estimation and evaluation. We have used approximately one fourth of our data set for model evaluation, which left us with only 14 seasons of training data and 5 seasons of testing data. Additionally, we had fewer than 14 seasons of leave-one-season-out predictions to use in estimating the model weighting functions for the **FW-wu** ensemble methods because the **SARIMA** model required unobserved seasonally lagged incidence to make predictions for the first few seasons in the training phase. This small sample size may have negatively impacted our ability to estimate the weighting functions. Altogether the test phase included 55 combinations of region and season, with a total of 2469 predictions from each method made across all three prediction targets before the test phase season onset or peak occurred. Nevertheless, because of the high degree of correlation in model log scores for the same prediction target in different weeks and regions within the same season we have a smaller effective sample size for detecting differences in average model performance in the test phase. The findings in this work should be confirmed with additional data sets. Another possible avenue would be to obtain pseudo out-of-sample results by performing cross validation within the training phase.

Another limitation of this work is the small selection of component models used. Theoretical results and applications have demonstrated that ensemble methods are most effective when using a diverse set of component models [16]. In our study, the **KCDE** and **SARIMA** component models are similar in that they both use seasonal terms and observations of recent incidence to inform their predictions (though we note that these two models tended to perform well in different seasons, as illustrated in S10 Fig). Increased component model diversity could yield improved ensemble performance; this could be achieved either through inclusion of different model structures (for example, agent-based or mechanistic models such as those explored in [5–10]) or different covariates (such as information about the circulating strains of a disease, spatial effects, weather, or social media data, as used by [5, 6, 8, 10–14]). Thus, the current work should not be viewed as a competitor to the models developed in previous work, but rather as a method for integrating and unifying the diverse array of methods that have been developed in the literature. The methods presented here are suitable for combining predictions from any collection of component models that each output a full predictive distribution, regardless of model structure.

Our exploration of feature-weighted ensembles is also limited by the relatively restricted feature sets we used for the weighting functions. We selected a few features based on exploratory analysis of the training phase results, and set all ensemble model formulations before obtaining any predictions for the test phase. It is possible that other weighting features not considered in this work may be more informative than those we have used. Some ideas for weighting covariates to use in future work include the largest incidence so far this season; the onset threshold; alternative summaries of the predictive distributions from the component models such as the probability at the mode or the modal value; the predominant flu strain; or the distribution of incidence in age groups.

The performance of the ensemble methods might be improved by subsetting the training data for the ensembles to the most important observations. The discrepancy in this work between the times used to train the ensembles (all leave-one-season-out predictions) and the times used for model comparison (only predictions made before the season onset or peak) may have led to an artificial decline in performance for the ensembles; this may be especially so for the relatively inflexible **CW** method.

This work provides a rigorous and comprehensive evaluation of ensemble methods for averaging probabilistic predictions for features of infectious disease outbreaks. A range of models, both single component models and ensemble models that combined component model predictions, demonstrated the ability to make more accurate predictions than a seasonal average baseline model. Additionally, systematic comparisons of simple and complex prediction models highlight a crucial added value of ensemble modeling, namely increased stability and consistency of model performance relative to the component models. Continued investigation, application, and innovation is necessary to strengthen our understanding of how to best leverage combinations of models to assist decision makers in fields, such as public health and infectious disease surveillance, that require data-driven rapid response.

## Supporting information

S1 Text Permutation test procedure. (PDF)

S1 Fig Predictive distributions for onset timing. Predictions are shown for just the FW-reg-w method at the national level, facetted by test phase season.
(PDF)

S2 Fig Predictive distributions for peak timing. Predictions are shown for just the FW-reg-w method at the national level, facetted by test phase season.
(PDF)

S3 Fig Predictive distributions for peak incidence. Predictions are shown for just the FW-reg-w method at the national level, facetted by test phase season.
(PDF)

S4 Fig Log scores achieved by each component model in each week of the season. For each week of the season, log scores are summarized across all seasons in the training phase when all three component models produced predictions. The thick line is a smoothed estimate of mean log score at each week in the season; the shaded region indicates the convex hull of log scores achieved by each model; and the actual log scores achieved in each week are indicated with points.
(PDF)

S5 Fig Log scores achieved by each component model vs. model uncertainty. Model uncertainty is measured by the number of bins required to cover 90% of the predictive distribution. The plot summarizes results across all seasons in the training phase when all three component models produced predictions. The thick line is a smoothed estimate of mean log score at each value of model uncertainty; the shaded region indicates the convex hull of log scores achieved by each model; and the actual log scores achieved in each week are indicated with points. The KCDE and SARIMA models condition on all previously observed data within the current season, and generally have high certainly when the target event (season onset or season peak) has almost occurred or has already occurred.
(PDF)

S6 Fig Log scores achieved by each component model vs. wILI in the week of the season when predictions were made. The plot summarizes results across all seasons in the training phase when all three component models produced predictions. The thick line is a smoothed estimate of mean log score at each week in the season; the shaded region indicates the convex hull of log scores achieved by each model; and the actual log scores achieved in each week are indicated with points.
(PDF)

S7 Fig Weights assigned to each component model by the FW-reg-wu ensemble model. Weights are shown for the prediction of season peak incidence at the national level. There are three weighting functions (one for each component model) represented in each row of the figure. The value of the weight is depicted by the color. Each function depends on three features: the week of the season at the time when the predictions are made, KCDE model uncertainty, and SARIMA model uncertainty. Model uncertainty represents the minimum number of predictive distribution bins required to cover 90% probability of the predictive distribution, so the higher this number is the more uncertain the model is.
(PDF)

S8 Fig Density plots representing the distribution of log score differences from predictions made by the FW-reg-w and SARIMA models. Predictions are aggregated across all regions and test phase seasons. The horizontal axis represents the difference in log scores achieved by the FW-reg-w and SARIMA models for predictions made in a particular week; positive values indicate that FW-reg-w outperformed SARIMA for that prediction. The vertical line indicates the mean log score difference for all predictions made before the onset or season peak occurred.
(PDF)

S9 Fig Permutation test results for pairwise comparisons of the 10th percentile of log score differences for each method relative to the median model. For each combination of 3 prediction targets, 11 regions, and 5 test phase seasons, we calculated the difference in mean log scores between each method and the method with median performance for that target, region, and season. Panel A presents the 10th percentile of these differences from the median model for each method across all combinations of target, region, and season. Larger values of this quantity indicate that the given model has better worst-case performance. Panel B displays the difference in this measure of worst-case performance for each pair of models. Positive values indicate that the model on the vertical axis had better worst-case performance than the model on the horizontal axis. A permutation test was used to obtain approximate p-values for these differences (see S1 Text for details). For reference, a Bonferroni correction at a familywise significance level of 0.05 for all pairwise comparisons leads to a significance cutoff of approximately 0.0014.
(PDF)

S10 Fig Model performance ranked by mean log score within each of the five test seasons. Only predictions made before the target (season onset or peak) occurred are included. Averages are taken across all regions.
(PDF)
