# Supplementary material for: Prediction of infectious disease epidemics via weighted density ensembles
Source: PLoS Comput Biol. 2018 Feb 20;14(2):e1005910. doi: 10.1371/journal.pcbi.1005910 (PMC5834190; doi:10.1371/journal.pcbi.1005910)
Supplement: S1 Text — (PDF) [file pcbi.1005910.s001.pdf]

S1 Text for Prediction of infectious disease epidemics via weighted density ensembles

Evan L. Ray and Nicholas G. Reich

S1 Text. Permutation Test Procedure

In the manuscript, we conducted permutation tests to compare mean performance and worst-case performance for different methods across all combinations of prediction target, region, and season. Here, we outline the procedure used for those permutation tests.

The first step in our analysis was to compute the mean log score for predictions made before the onset week (for predictions of onset timing) or the peak week (for predictions of peak timing or peak incidence). As discussed in the manuscript, this ensures that results for seasons with early onset times count with equal weight as seasons with late onset times in model comparisons. It also means that the permutation test procedure may lose power that would be available from comparing results in individual weeks; however, due to the presence of serial auto-correlation in model performance in consecutive weeks, we surmised that the loss in power would not be very dramatic. After this step, we have 165 measures of mean performance for each of our 9 models: one for each combination of the 3 prediction targets, 11 spatial units, and 5 test phase seasons. These measures of mean performance were used directly in permutation tests for overall average performance. For tests of worst-case performance, we calculated the difference between the mean performance for a given model, target, region, and season and the median performance over all models we considered for that target, region, and season.

These computations give a score $\tau_{m,p,r,s}$, for each combination of model m, prediction target p, region r, and test phase season s (where which score is used depends on whether we are testing mean performance or worst-case performance). Denote the vector of these scores for a particular model by τ_m. To compare these values for a given pair of models m_1 and m_2, the observed test statistic is $|\text{mean}(\tau_{m_1}) - \text{mean}(\tau_{m_2})|$ for comparisons of mean performance and $|\text{min}(\tau_{m_1}) - \text{min}(\tau_{m_2})|$ for comparisons of worst-case performance.

The permutation test evaluated whether the scores $\tau_{m_1,p,r,s}$ and $\tau_{m_2,p,r,s}$ were drawn from the same distribution within each combination of p, r, and s. Specifically for each each combination of values p, r and s, we permuted the values of $\tau_{m_1,p,r,s}$ and $\tau_{m_2,p,r,s}$. This yields a new pair of permuted vectors $\tilde{\tau}_{m_1}$ and $\tilde{\tau}_{m_2}$ and a corresponding test statistic value. Repeating the permutation process 100,000 times yielded an approximate sampling distribution for the test statistic under the null hypothesis, from which we calculated an approximate p-value.

We note that the "paired permutation" strategy presented here accounts for the fact that some prediction targets, regions, and seasons are more difficult to predict than others, so scores are not exchangeable across different combinations of those factors. However, this procedure does not capture possible correlation in the performance of a single model across different regions within a given season or different seasons within a given region.
