# Supplementary material for: Prediction of infectious disease epidemics via weighted density ensembles
Source: PLoS Comput Biol. 2018 Feb 20;14(2):e1005910. doi: 10.1371/journal.pcbi.1005910 (PMC5834190; doi:10.1371/journal.pcbi.1005910)
Supplement: S3 Fig — Predictions are shown for just the FW-reg-w method at the national level, facetted by test phase season. (PDF) [file pcbi.1005910.s004.pdf]

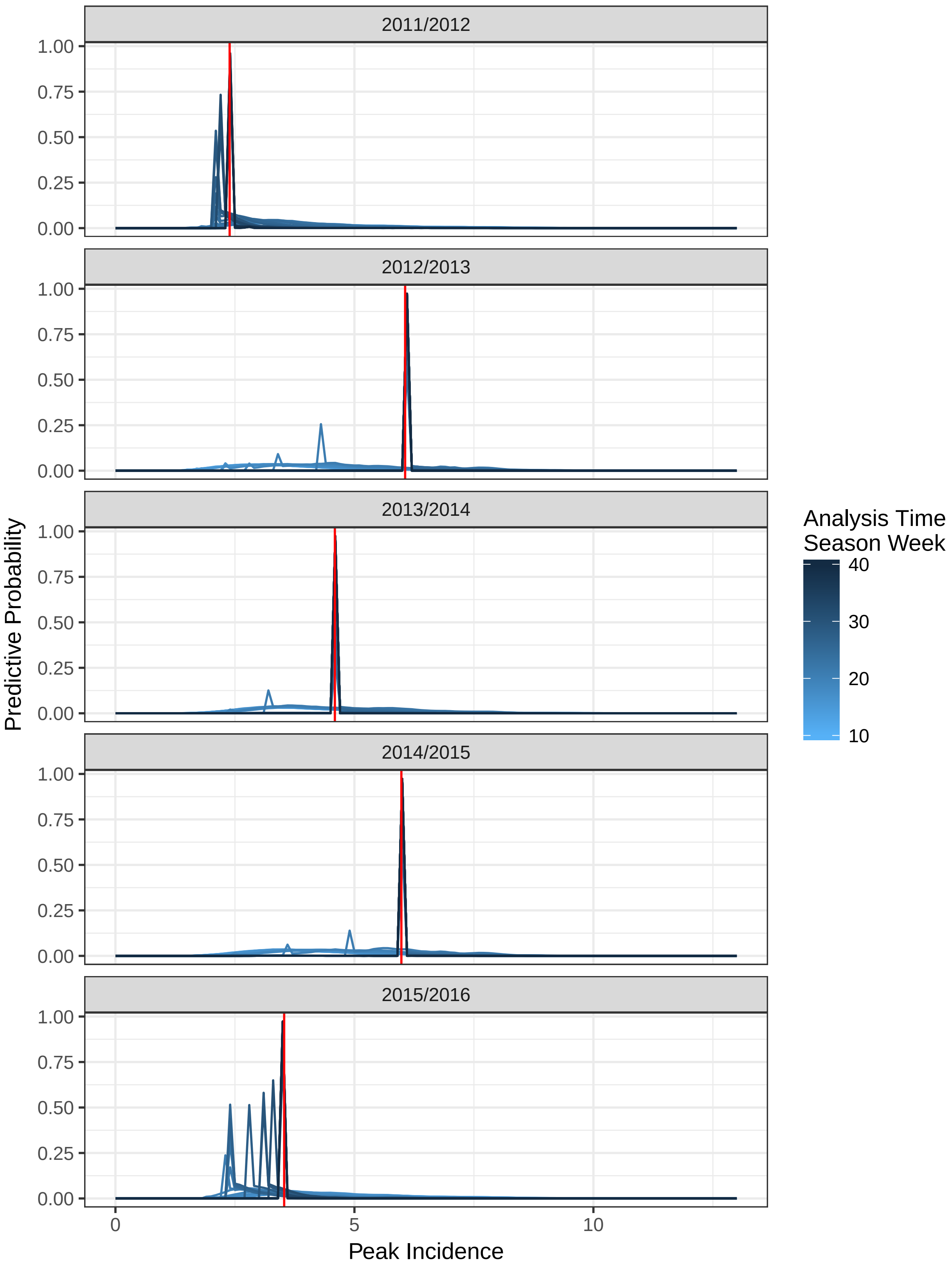

S3 Fig. Predictive distributions for peak incidence. Predictions are shown for just the FW-reg-w method at the national level, facetted by test phase season.
