# Supplementary material for: Prediction of infectious disease epidemics via weighted density ensembles
Source: PLoS Comput Biol. 2018 Feb 20;14(2):e1005910. doi: 10.1371/journal.pcbi.1005910 (PMC5834190; doi:10.1371/journal.pcbi.1005910)
Supplement: S5 Fig — Model uncertainty is measured by the number of bins required to cover 90% of the predictive distribution. The plot summarizes results across all seasons in the training phase when all three component models produced predictions. The thick line is a smoothed estimate of mean log score at each value of model uncertainty; the shaded region indicates the convex hull of log scores achieved by each model; and the actual log scores achieved in each week are indicated with points. The KCDE and SARIMA models condition on all previously observed data within the current season, and generally have high certainly when the target event (season onset or season peak) has almost occurred or has already occurred. (PDF) [file pcbi.1005910.s006.pdf]

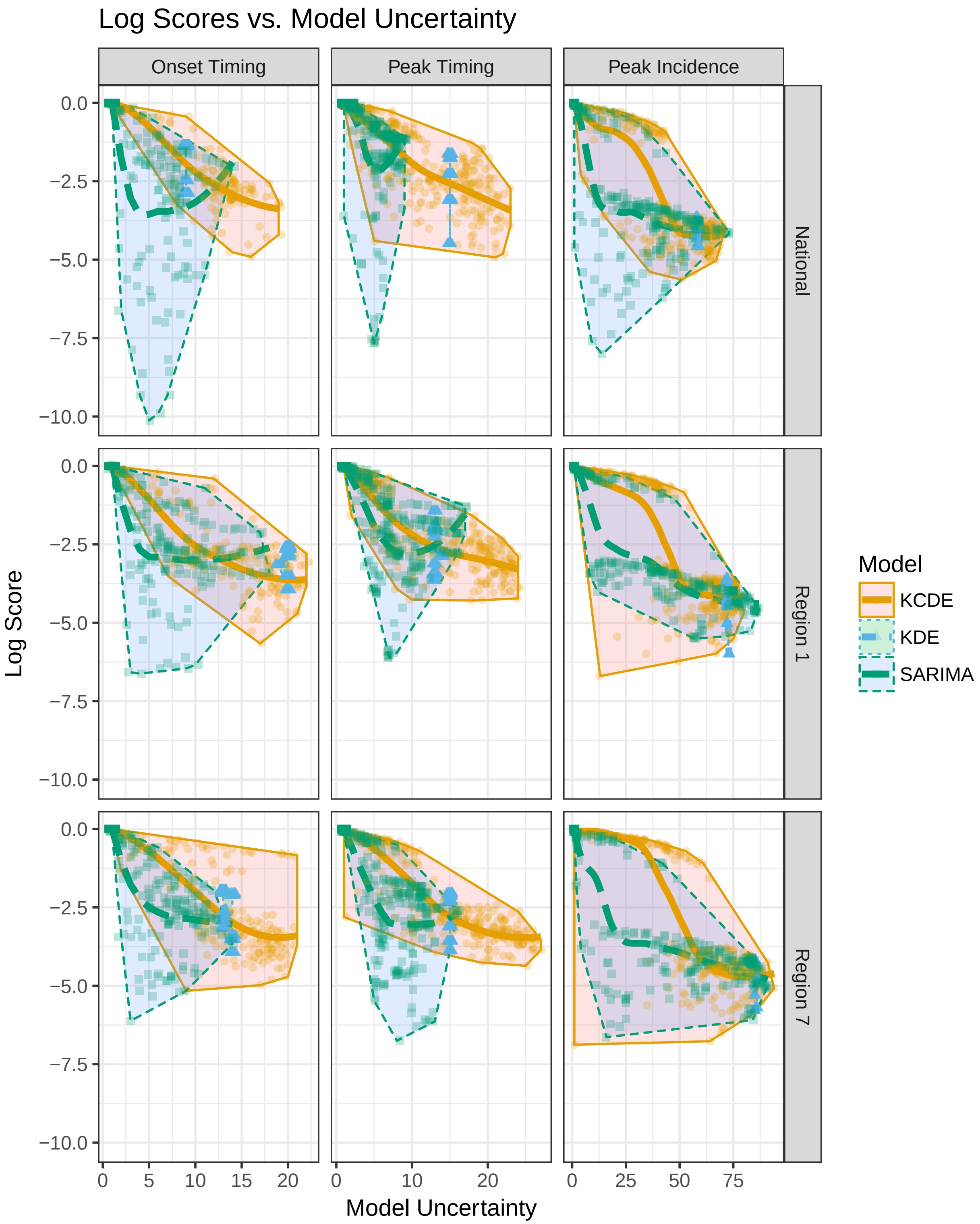

S5 Fig. Log scores achieved by each component model vs. model uncertainty Model uncertainty is measured by the number of bins required to cover 90% of the predictive distribution. The plot summarizes results across all seasons in the training phase when all three component models produced predictions. The thick line is a smoothed estimate of mean log score at each value of model uncertainty; the shaded region indicates the convex hull of log scores achieved by each model; and the actual log scores achieved in each week are indicated with points. The KCDE and SARIMA models condition on all previously observed data within the current season, and generally have high certainly when the target event (season onset or season peak) has almost occurred or has already occurred.
