# Supplementary material for: Prediction of infectious disease epidemics via weighted density ensembles
Source: PLoS Comput Biol. 2018 Feb 20;14(2):e1005910. doi: 10.1371/journal.pcbi.1005910 (PMC5834190; doi:10.1371/journal.pcbi.1005910)
Supplement: S6 Fig — The plot summarizes results across all seasons in the training phase when all three component models produced predictions. The thick line is a smoothed estimate of mean log score at each week in the season; the shaded region indicates the convex hull of log scores achieved by each model; and the actual log scores achieved in each week are indicated with points. (PDF) [file pcbi.1005910.s007.pdf]

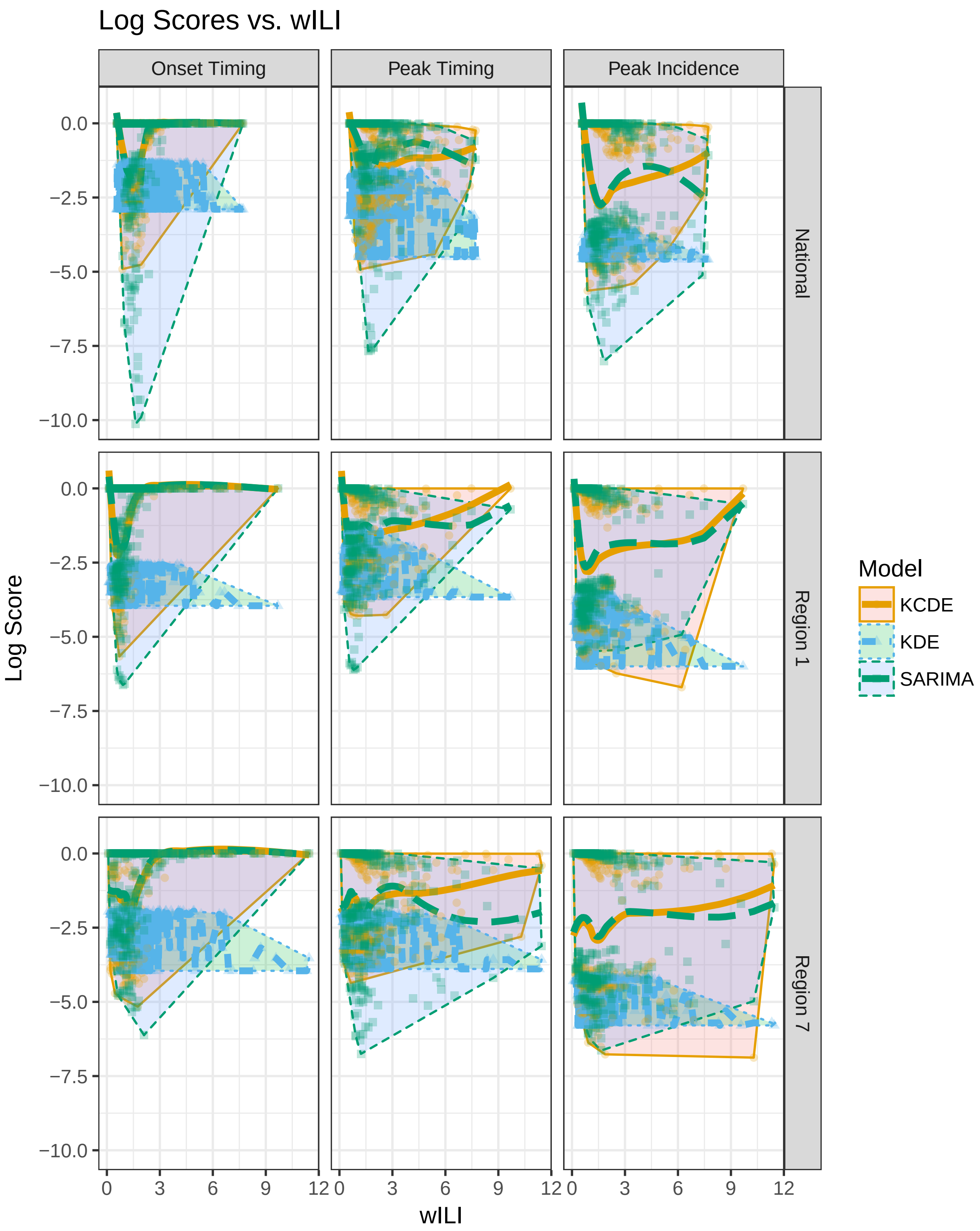

S6 Fig. Log scores achieved by each component model vs. wILI in the week of the season when predictions were made. The plot summarizes results across all seasons in the training phase when all three component models produced predictions. The thick line is a smoothed estimate of mean log score at each week in the season; the shaded region indicates the convex hull of log scores achieved by each model; and the actual log scores achieved in each week are indicated with points.
