# Supplementary material for: Prediction of infectious disease epidemics via weighted density ensembles
Source: PLoS Comput Biol. 2018 Feb 20;14(2):e1005910. doi: 10.1371/journal.pcbi.1005910 (PMC5834190; doi:10.1371/journal.pcbi.1005910)
Supplement: S7 Fig — Weights are shown for the prediction of season peak incidence at the national level. There are three weighting functions (one for each component model) represented in each row of the figure. The value of the weight is depicted by the color. Each function depends on three features: the week of the season at the time when the predictions are made, KCDE model uncertainty, and SARIMA model uncertainty. Model uncertainty represents the minimum number of predictive distribution bins required to cover 90% probability of the predictive distribution, so the higher this number is the more uncertain the model is. (PDF) [file pcbi.1005910.s008.pdf]

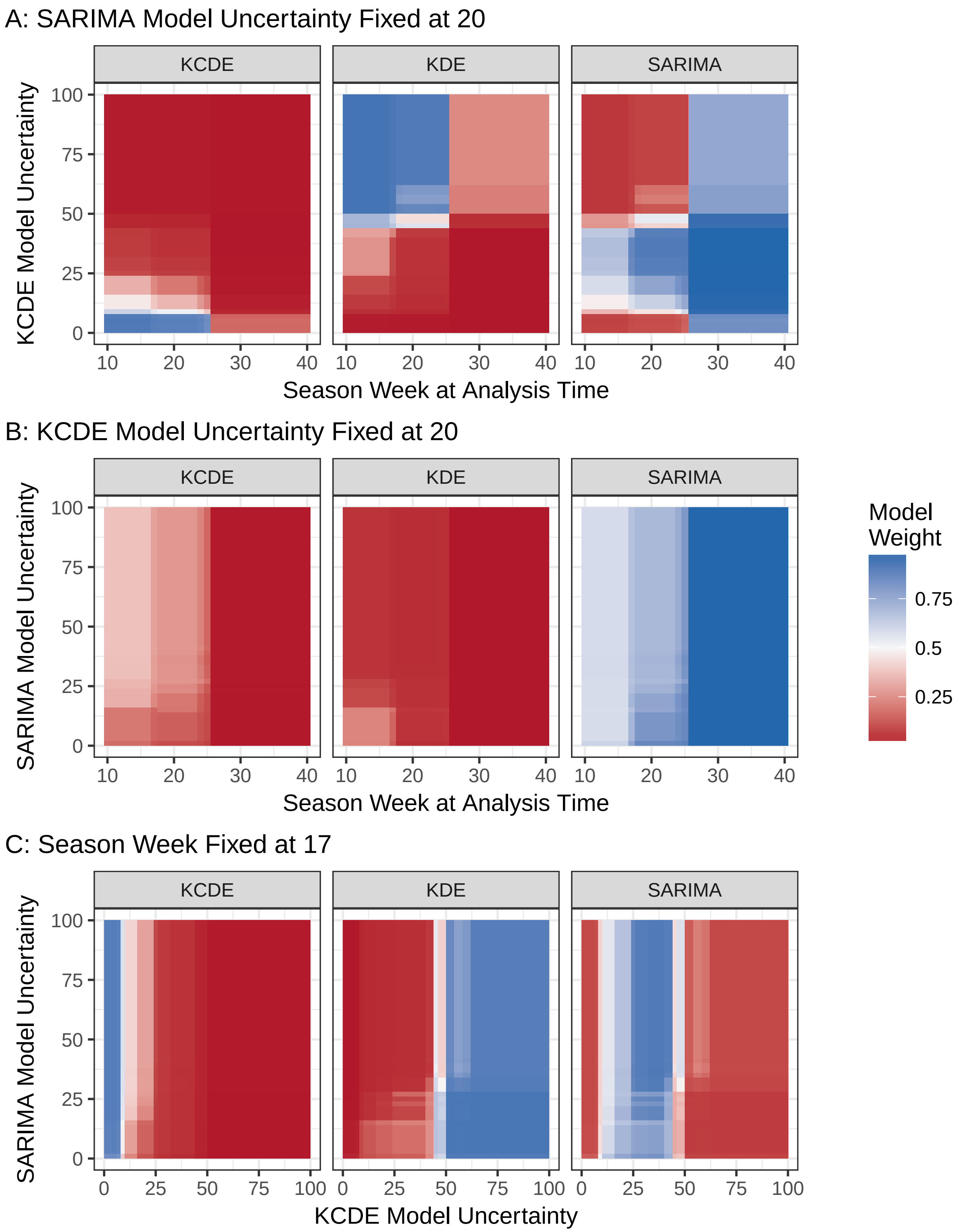

S7 Fig. Weights assigned to each component model by the FW-reg-wu ensemble model. Weights are shown for the prediction of season peak incidence at the national level. There are three weighting functions (one for each component model) represented in each row of the figure. The value of the weight is depicted by the color. Each function depends on three features: the week of the season at the time when the predictions are made, KCDE model uncertainty, and SARIMA model uncertainty. Model uncertainty represents the minimum number of predictive distribution bins required to cover 90% probability of the predictive distribution, so the higher this number is the more uncertain the model is.
