# Supplementary material for: Prediction of infectious disease epidemics via weighted density ensembles
Source: PLoS Comput Biol. 2018 Feb 20;14(2):e1005910. doi: 10.1371/journal.pcbi.1005910 (PMC5834190; doi:10.1371/journal.pcbi.1005910)
Supplement: S8 Fig — Predictions are aggregated across all regions and test phase seasons. The horizontal axis represents the difference in log scores achieved by the FW-reg-w and SARIMA models for predictions made in a particular week; positive values indicate that FW-reg-w outperformed SARIMA for that prediction. The vertical line indicates the mean log score difference for all predictions made before the onset or season peak occurred. (PDF) [file pcbi.1005910.s009.pdf]

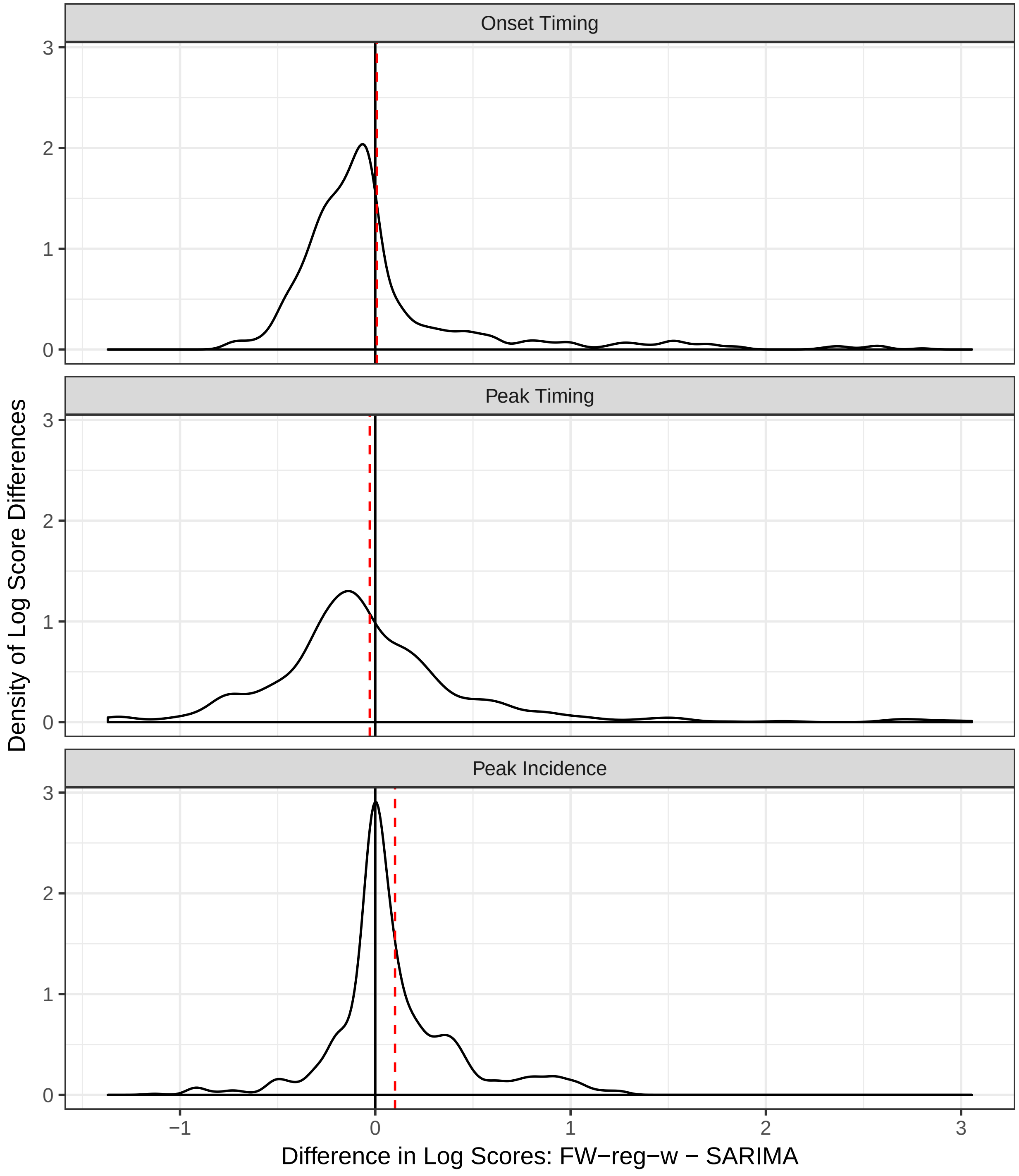

S8 Fig. Density plots representing the distribution of log score differences from predictions made by the FW-reg-w and SARIMA models. Predictions are aggregated across all regions and test phase seasons. The horizontal axis represents the difference in log scores achieved by the FW-reg-w and SARIMA models for predictions made in a particular week; positive values indicate that FW-reg-w outperformed SARIMA for that prediction. The vertical line indicates the mean log score difference for all predictions made before the onset or season peak occurred.
