# Supplementary material for: Prediction of infectious disease epidemics via weighted density ensembles
Source: PLoS Comput Biol. 2018 Feb 20;14(2):e1005910. doi: 10.1371/journal.pcbi.1005910 (PMC5834190; doi:10.1371/journal.pcbi.1005910)
Supplement: S9 Fig — For each combination of 3 prediction targets, 11 regions, and 5 test phase seasons, we calculated the difference in mean log scores between each method and the method with median performance for that target, region, and season. Panel A presents the 10th percentile of these differences from the median model for each method across all combinations of target, region, and season. Larger values of this quantity indicate that the given model has better worst-case performance. Panel B displays the difference in this measure of worst-case performance for each pair of models. Positive values indicate that the model on the vertical axis had better worst-case performance than the model on the horizontal axis. A permutation test was used to obtain approximate p-values for these differences (see S1 Text for details). For reference, a Bonferroni correction at a familywise significance level of 0.05 for all pairwise comparisons leads to a significance cutoff of approximately 0.0014. (PDF) [file pcbi.1005910.s010.pdf]

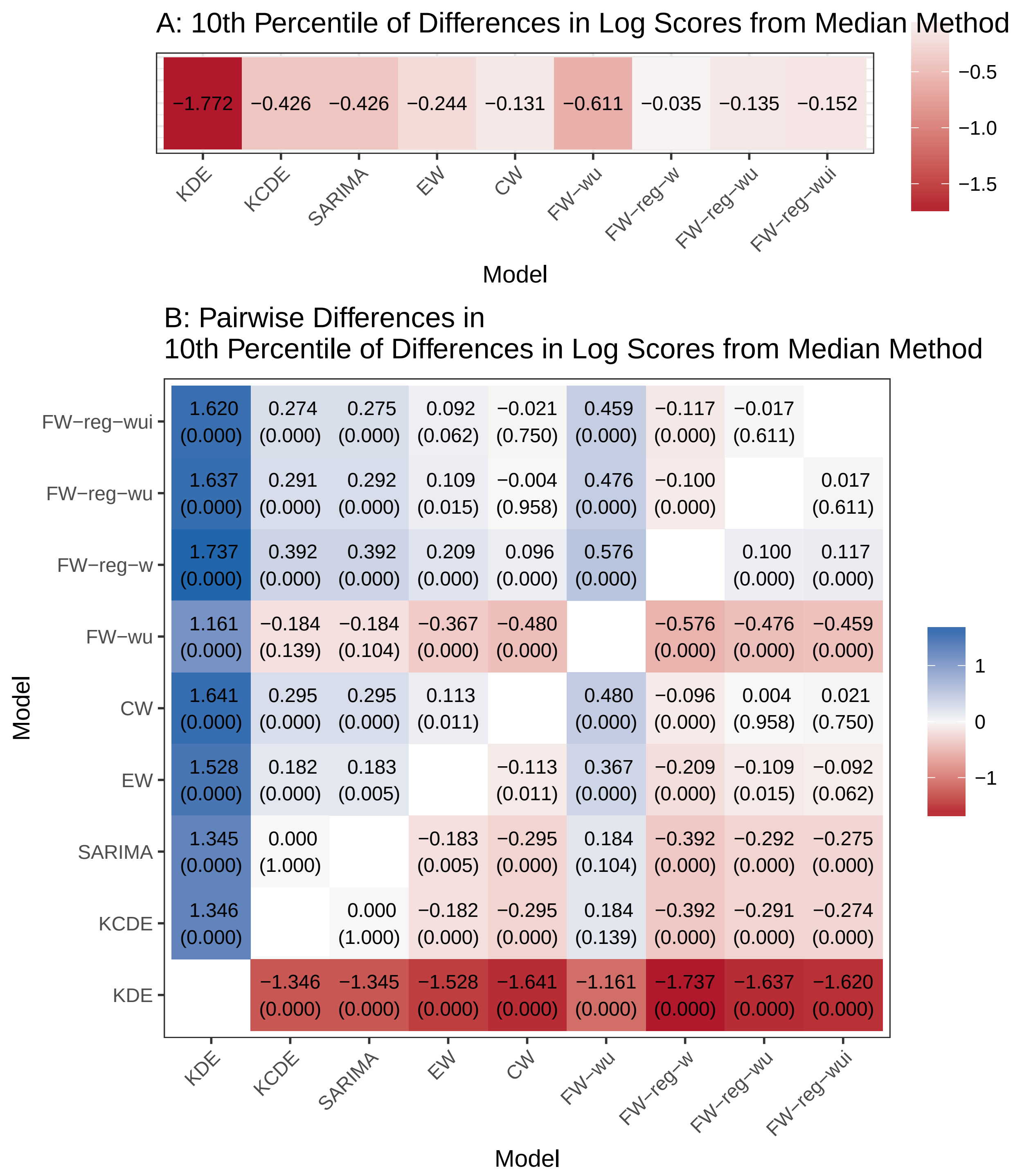

S9 Fig. Permutation test results for pairwise comarisons of the 10th percentile of log score differences for each method relative to the median model. For each combination of 3 prediction targets, 11 regions, and 5 test phase seasons, we calculated the difference in mean log scores between each method and the method with median performance for that target, region, and season. Panel A presents the 10th percentile of these differences from the median model for each method across all combinations of target, region, and season. Larger values of this quantity indicate that the given model has better worst-case performance. Panel B displays the difference in this measure of worst-case performance for each pair of models. Positive values indicate that the model on the vertical axis had better worst-case performance than the model on the horizontal axis. A permutation test was used to obtain approximate p-values for these differences (see supplement for details). For reference, a Bonferroni correction at a familywise significance level of 0.05 for all pairwise comparisons leads to a significance cutoff of approximately 0.0014.
