# Supplementary material for: Prediction of infectious disease epidemics via weighted density ensembles
Source: PLoS Comput Biol. 2018 Feb 20;14(2):e1005910. doi: 10.1371/journal.pcbi.1005910 (PMC5834190; doi:10.1371/journal.pcbi.1005910)
Supplement: S10 Fig — Only predictions made before the target (season onset or peak) occurred are included. Averages are taken across all regions. (PDF) [file pcbi.1005910.s011.pdf]

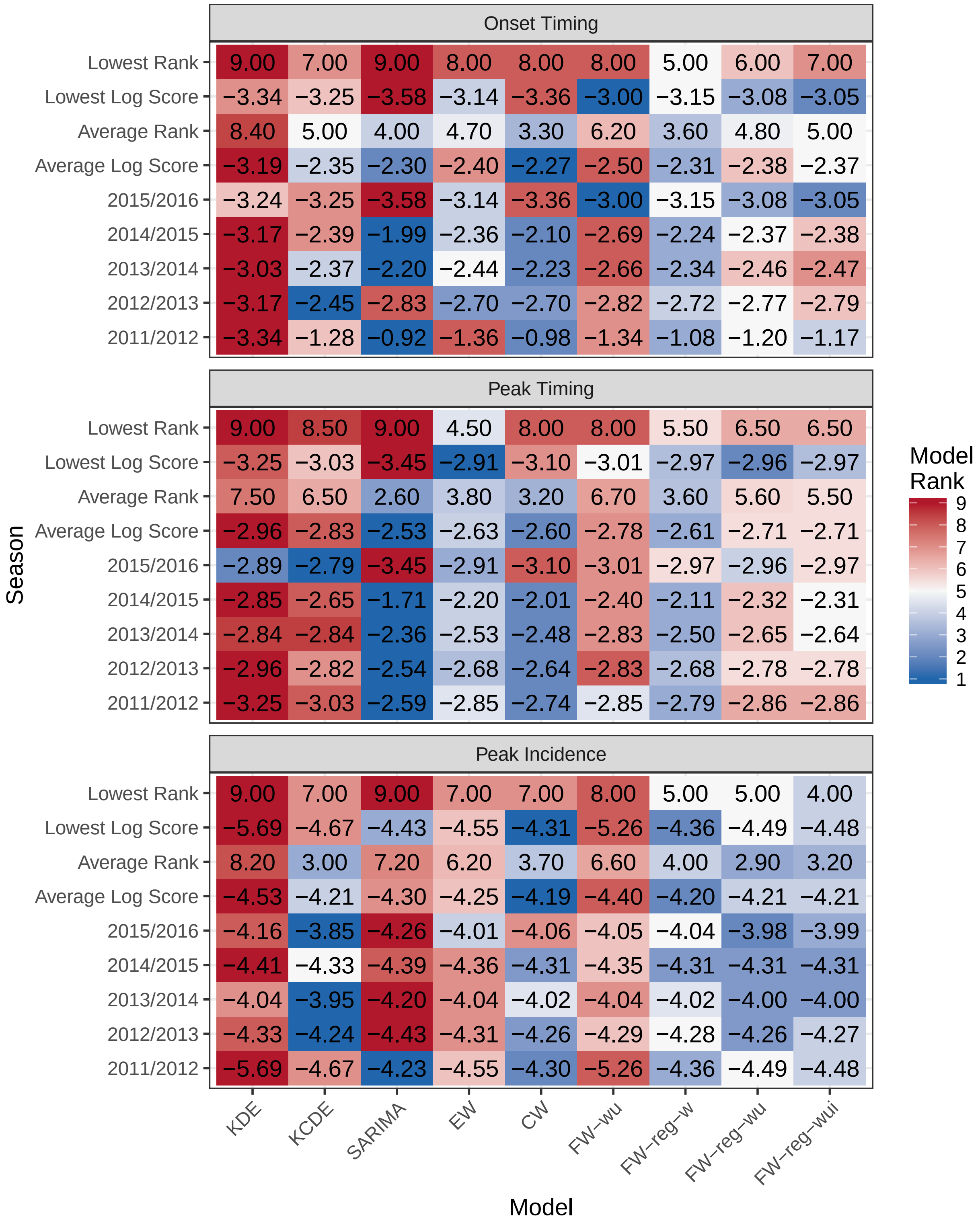

S10 Fig. Model performance ranked by mean log score within each of the five test seasons. Only predictions made before the target (season onset or peak) occurred are included. Averages are taken across all regions.
